# Genome-Wide Identification and Functional Analysis of the *CNGC* Gene Family in *Suaeda glauca*

**DOI:** 10.3390/biology15060467

**Published:** 2026-03-13

**Authors:** Jun Wang, Chunxing Dong, Xiaoxue He, Dongpeng Zheng, Xingguang Chen, Jiahao Cai, Gang Wang, Boping Tang, Chunyin Zhang, Lulu Wang, Xiaoping Niu, Chunmei Lai, Yuan Qin, Yan Cheng

**Affiliations:** 1 College of Life Science, State Key Laboratory of Agricultural and Forestry Biosecurity, College of Plant Protection, Fujian Provincial Key Laboratory of Haixia Plant Systems Biology, School of Future Technology, Haixia Institute of Science and Technology, School of Future Technology, Fujian Agriculture and Forestry University, Fuzhou 350002, China; 15345915026@163.com (J.W.); d_chunxing@163.com (C.D.); hexiaoxue@ynby.cn (X.H.); 13546825440@163.com (D.Z.); chen_xg0722@163.com (X.C.); m13727960641@163.com (J.C.); luluwanghn@163.com (L.W.); xpniu0613@126.com (X.N.); cmlai21@fafu.edu.cn (C.L.); 2 Jiangsu Key Laboratory for Bioresources of Saline Soils, Yancheng Teachers University, Yancheng 224051, China; baiwang0708@163.com (G.W.); boptang@163.com (B.T.); 3 Yancheng Lvyuan Salt Soil Agricultural Technology Co., Ltd., Yancheng 224051, China; chinahpz@163.com

**Keywords:** *Suaeda glauca*, CNGC gene family, salt stress

## Abstract

Cyclic nucleotide-gated channel (*CNGC*) genes play key regulatory roles in plant immunity and abiotic stress responses. In this study, we conducted a genome-wide identification and analysis of the *CNGC* gene family in *Suaeda glauca*. A total of 44 *SgCNGC* genes were identified. Through phylogenetic analysis, gene structure analysis, chromosome distribution, conserved motif analysis, collinearity analysis, cis-acting element analysis, subcellular localization, and gene overexpression analysis, we systematically characterized the evolutionary relationships, structural features, and potential functions of this gene family. The results indicate that the *SgCNGC* gene family is evolutionarily highly conserved but exhibits functional divergence in structure and expression. Furthermore, functional assays revealed that overexpression of SgCNGC13 in Arabidopsis thaliana led to increased salt sensitivity, indicating a negative regulatory role for this gene under salt stress. These findings provide a foundation for understanding the role of the *CNGC* gene family in the growth, development, and stress response of *S. glauca* and contribute to the remediation of saline–alkali land.

## Simple Summary

Soil salinization is a growing problem that is harming agriculture and the environment. *Suaeda glauca* is a plant that grows in saline–alkali land, but the genetic regulatory mechanisms underlying its salt tolerance are not fully understood. This study explores a family of genes, Cyclic nucleotide-gated channel genes, that play key roles in helping plants cope with stress. A total of 44 members of this gene family were identified in *Suaeda glauca*, and their characteristics were analyzed. We found that when the expression of one gene was significantly increased in *Suaeda glauca*, the plant became more sensitive to salt, suggesting that this gene may negatively regulate salt tolerance, which provides a basis for understanding how the plant survives in high-salt environments. In the future, these findings may help scientists develop new crop varieties that are better suited to growing in saline–alkali soils, aiding in food security and the restoration of degraded lands.

## 1. Introduction

The salinized land area in China accounts for 10.1% of the global total [1], making it the country with the largest area of soil salinization [2]. As potential reserve arable land resources, these lands hold significant development potential. Soil salinization is primarily caused by natural factors (e.g., evaporation exceeding precipitation in arid regions, seawater intrusion in coastal areas) and human activities (e.g., improper irrigation, excessive fertilizer application), often leading to soil compaction, decreased fertility, and ecological degradation. It has become a key constraint on the sustainable development of agriculture in China. *Suaeda glauca* (*S. glauca*) is an annual herb belonging to the family Chenopodiaceae and genus Suaeda. Morphologically, its succulent leaves and stems contribute to reduced transpiration and enhanced water storage capacity. Additionally, its cells exhibit strong osmotic potential, enabling tolerance to high-salt environments. It is widely distributed in coastal areas of China, playing an important role in the ecological restoration of saline–alkali land and the maintenance of biodiversity [3].

Cyclic nucleotide-gated channel (*CNGC*) genes are a type of calcium ion transport channels, generally composed of six transmembrane (TM) domains, calmodulin binding domains (CaMBDs), and cyclic nucleotide-binding domains (CNBDs). Among them, the CNBD domain is highly conserved and can specifically recognize and bind to cyclic nucleotide molecules [4]. Ca^2+^, as an important second messenger, is widely involved in regulating plant growth, development, and responses to abiotic stresses (such as osmotic and ionic stress) [5]. *CNGCs* are widely present in the plant kingdom (e.g., rapeseed, maize, tomato, potato) and function as calcium ion channels in plants, participating in signal transduction of various physiological processes by modulating calcium ion concentration. The number of *CNGC* genes and their distribution across clades vary among different plants, and their functions also differ. For example, Chen et al. identified 49 *BnCNGC* genes in *Brassica napus*, among which *BnCNGC9* from Group IVa and *BnCNGC27* and *BnCNGC48* from Group IVb exhibited different expression patterns under various stress conditions [6].

In recent years, numerous studies have reported on gene families in *Suaeda* and the *CNGC* gene family in plants. Wang et al. [7] found that AGO proteins play an important role in salt stress response in *Suaeda salsa*. Furthermore, Baloch [8] and Zhang et al. [9] reported significant associations of this gene family with salt stress and drought tolerance in alfalfa and transgenic *A. thaliana*, respectively. However, there have been no reports on the genome-wide identification and systematic analysis of the *CNGC* gene family in *S. glauca* to date. This study utilized bioinformatics approaches to analyze the members, physicochemical properties, subcellular localization, and phylogenetic relationships of the *CNGC* gene family in *S. glauca*, aiming to provide a theoretical basis for further investigation into the functions of *S. glauca* gene families, screening of stress-resistant varieties, and promoting its application in saline–alkali land.

## 2. Materials and Methods

### 2.1. Experimental Materials

The *S. glauca* cultivar used in this study were provided by Yancheng Lvyuan Saline Soil Agriculture Technology Co., Ltd., Yancheng, Jiangsu, Southeast China (http://www.ychpz.com/, accessed on 15 September 2025). The cultivation of *S. glauca* followed that of Mohammadi et al. [10]. *Nicotiana benthamiana* (*N. benthamiana*) seeds were sourced from the laboratory seed bank and planted in a mixed substrate (vermiculite: nutrient soil = 1:1) at 28 °C. *Arabidopsis thaliana* Col-0 plants obtained from ABRC (Arabidopsis Biological Resources Center, https://abrc.osu.edu/, accessed on 15 September 2025) were used as the wild type and cultured on 1/2 MS medium at 25 °C.

### 2.2. Analysis of Protein Physicochemical Properties

Twenty CNGC protein sequences from *Arabidopsis thaliana* were retrieved from the Arabidopsis Information Resource (TAIR10) database (http://www.arabidopsis.org/, accessed on 20 September 2025). The genome of *S. glauca* was obtained from China National Center for Bioinformation (https://www.cncb.ac.cn/, accessed on 20 September 2025) under the accession number WGS038631 [11]. Using this information, BLASTp alignment was performed, combined with domain prediction using the SMART online tool (http://smart.embl-heidelberg.de/, accessed on 22 September 2025) [12]. Based on the presence of TM domains, CaMBD, and CNBD structures and manually removing redundant sequences, *CNGC* genes in *S. glauca* were identified. The online tool ExPASy ProtParam (https://web.expasy.org/protparam/, accessed on 22 September 2025) was used to analyze physicochemical properties such as the isoelectric point, number of amino acids, and molecular weight of the *CNGC* family members. The subcellular localization of SgCNGC proteins was predicted using the online software CELLO (https://cello.life.nctu.edu.tw/, accessed on 22 September 2025) [13].

### 2.3. Phylogenetic, Conserved Domain, and Sequence Alignment Analysis of Gene Families

Phylogenetic analysis of *CNGC* gene family members from *Arabidopsis thaliana* and *S. glauca* was performed using MEGA11.0 (https://www.megasoftware.net/, accessed on 22 September 2025). The maximum likelihood (ML) method [14] was employed with the JTT+G (Jones-Taylor-Thornton + Gamma distribution) model, and bootstrap support values were calculated from 1000 replicates. The Evolview online platform (https://www.evolgenius.info/evolview/, accessed on 25 September 2025) was used for further visualization and refinement of the phylogenetic tree [15]. The CNGC protein sequences from *A. thaliana* and *S. glauca* were imported into MEGA11.0 software for sequence alignment using the Muscle algorithm [16] to identify conserved domains, and the results were exported in FASTA format. Jalview 2 software was used for final visualization [17].

### 2.4. Chromosomal Localization and Collinearity Analysis of the Gene Family

TBtools software (v2.315) was used to analyze chromosomal localization of *SgCNGC* genes and to generate chromosomal distribution maps. Protein-coding genes for *A. thaliana*, *Oryza sativa* (*O. sativa*), *Vitis vinifera* (*V. vinifera*), *Ipomoea batatas* (*I. batatas*)*,* and *Solanum lycopersicum* (*S. lycopersicum*) were downloaded from the public database EnsemblPlants (http://plants.ensembl.org/index.html, accessed on 25 September 2025). The multiple collinearity scan tool MCScanX was used to analyze the collinearity of *CNGC* genes between the *S. glauca* genome sequence and other species [18]. TBtools software was used for visualization, constructing intra-species collinearity circos plots and inter-species collinearity maps.

### 2.5. Analysis of Gene Structure and Cis-Acting Elements of Gene Family

The protein amino acid sequences of SgCNGC proteins were uploaded to MEME (https://meme-suite.org/, accessed on 26 September 2025) for motif analysis. The number of motifs was set to 10, with other parameters as default [19]. TBtools software was used for motif visualization. The upstream 2000 bp sequences of the transcription start site of *SgCNGC* genes extracted by TBtools were considered as the putative promoter region and were used to predict upstream cis-regulatory elements with the PlantCARE database (https://bioinformatics.psb.ugent.be/webtools/plantcare/html/, accessed on 26 September 2025) [20], and the results were visualized using TBtools software.

### 2.6. Protein Subcellular Localization

The cDNA samples of *S. glauca* were use as a template for amplifying the coding DNA sequence (CDS) sequence of *SgCNGC1*3 [21]. The pCAMBIA2300-SgCNGC13-GFP fusion expression vector was constructed by homologous recombination. The resulting constructs were transiently expressed in 4-week-old *N. benthamiana* leaves via *Agrobacterium*-mediated transformation. After 24 h of transformation, the *N. benthamiana* leaf disks were observed under a Leica TCS SP8X confocal microscope [22].

### 2.7. Analysis of Transgenic Overexpression Lines

Base deletions and insertions were occurred in the sequence alignment of SgCNGC13, suggesting that its function might be altered. Therefore, it was selected for further functional verification. The CDS of *SgCNGC13* was cloned into the pGWB505 binary vector using double digestion (XhoI and SalI) and the ligation cloning method. *A. thaliana* transformation was performed via the *Agrobacterium*-mediated floral dip method [23]. Transgenic seeds were screened on 1/2 MS medium (pH 5.8) containing 50 mg L^−1^ hygromycin. The T3 homozygous transgenic lines with high *SgCNGC13* expression levels and wild-type *A. thaliana* seeds were sterilized and germinated on 1/2 MS plates supplemented with 0, 75, 100, or 150 mM NaCl, and cultured at 25 °C under a 16/8 h light/dark cycle. Root lengths were observed and measured after 2 weeks to evaluate their salt tolerance potential. For statistical analysis, root length measurements were collected from at least 15 seedlings per genotype per treatment, with three independent biological replicates. Data were expressed as mean ± standard deviation (SD). Statistical analyses were performed using GraphPad Prism software 10.1.2.

## 3. Results

### 3.1. Genome-Wide Identification and Physicochemical Property Analysis of CNGC Gene Family in S. glauca

44 non-redundant genes encoding CNGC proteins were identified from the *S. glauca* genome (Supplementary Table S1). Based on their chromosomal distribution [24] and the similarity to *A. thaliana* homologues, the *CNCG* genes in *S. glauca* were renamed *SgCNGC1* to *SgCNGC44*. The encoded proteins of the *SgCNGCs* range in length from 411 to 1734 aa, with molecular weights from 47.60 to 194.87 kDa and aliphatic index from 80.72 to 107.66. Subcellular localization prediction indicated that the plasma membrane is the most consistently predicted location for most members.

### 3.2. Phylogenetic Analysis Showed a Close Genetic Relationship and Similarity Between S. glauca and A. thaliana CNGC Genes

**Figure 1:**
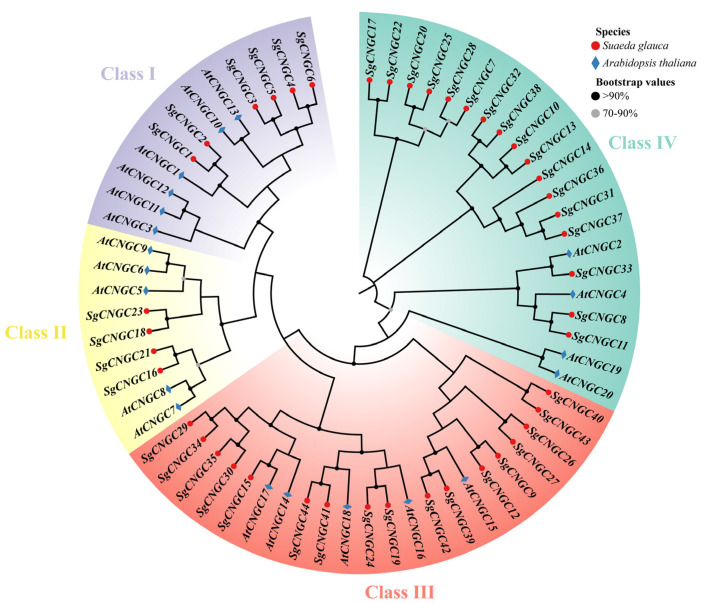
**Phylogenetic tree of the CNGC gene family of *Suaeda glauca* and *Arabidopsis thaliana*.** Purple, yellow, red and green represent Class I, II, III and IV, respectively. Red dots represent the *SgCNGC* gene family; and blue squares represent the *AtCNGC* gene family. Black dots on branches indicate bootstrap values >90%, and grey dots indicate values between 70% and 90%.

The maximum likelihood phylogenetic tree clearly divided the 44 *SgCNGC* genes into four subgroups (Class I, II, III, IV), with a relatively even distribution of members across branches and no aggregation of identical protein genes (Figure 1). Bootstrap support values for most nodes were above 90%, with some between 70 and 90%, indicating a reliable topological structure of the phylogenetic tree. Phylogenetic analysis indicated a close genetic relationship and similarity between *S. glauca* and *A. thaliana CNGC* genes.

### 3.3. Analysis of Conserved Domains and Sequence Alignment of the S. glauca CNGC Gene Family

**Figure 2:**
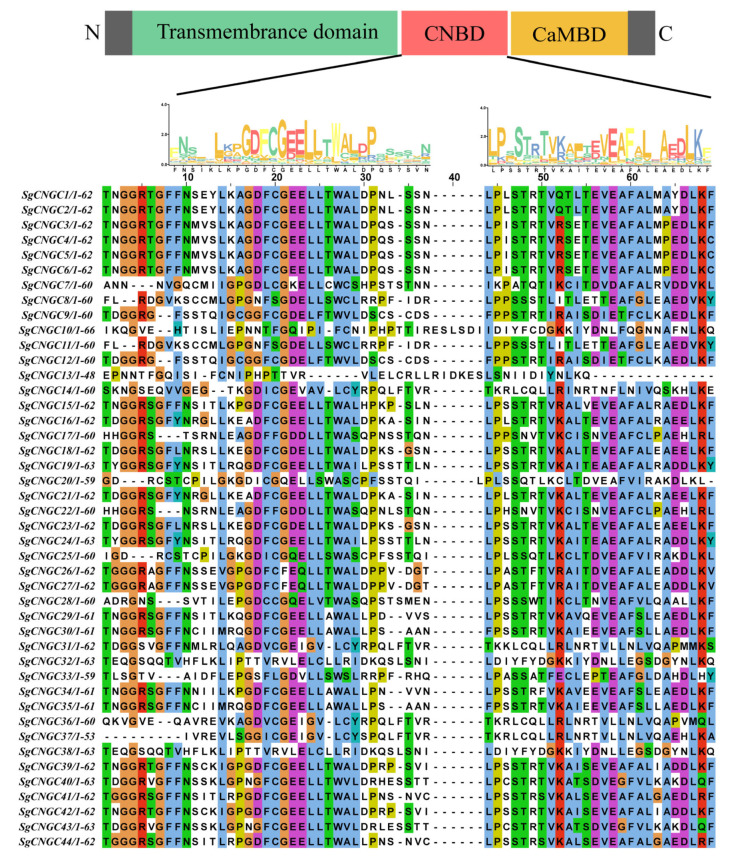
**Conserved domains and sequence alignment of the *Suaeda glauca* CNGC gene family.** Different colors represent amino acids with different chemical properties (for example, hydrophobic, polar, basic, acidic). The sequence logo at the top was generated by the MEME, which illustrates the conservation of residues within specific motifs in the CNBD domain, while the height of each letter is proportional to the frequency of the corresponding residue at that position.

Sequence alignment results (Figure 2) show that all members of the *S. glauca CNGC* gene family possess CaMBD and CNBD domains at the carboxyl terminus (C-terminus) and transmembrane domains at the amino terminus (N-terminus), which are characteristic of typical plant *CNGC* gene families [25]. Motif analysis identified conserved motifs specific to the *S. glauca CNGC* genes in the CNBD, with glycine (G) and leucine (L) appearing most frequently as the most conserved residues. These residues are crucial in maintaining structural stability, cyclic nucleotide binding, and ion channel regulation in CNGC proteins. Notably, *SgCNGC10*, *SgCNGC13*, *SgCNGC14*, *SgCNGC20*, *SgCNGC31*, *SgCNGC32*, *SgCNGC36*, *SgCNGC37*, and *SgCNGC38*, located in subgroup IV, exhibited a high number of non-conserved residues in the CNBD domain. *SgCNGC10* and *SgCNGC13* contained unique insertion sequences, while *SgCNGC13* also exhibited a deletion of only 48 amino acid residues. Overall, members of gene subgroups Class I and II showed extremely high sequence similarity in the CNBD region, with better conservation than Class III and Class IV. The conserved and variable patterns of *S. glauca CNGC* gene sequences were highly consistent with the classification in the phylogenetic tree (Figure 1).

### 3.4. Chromosomal Localization and Collinearity Comparative Analysis of the S. glauca CNGC Gene Family

**Figure 3:**
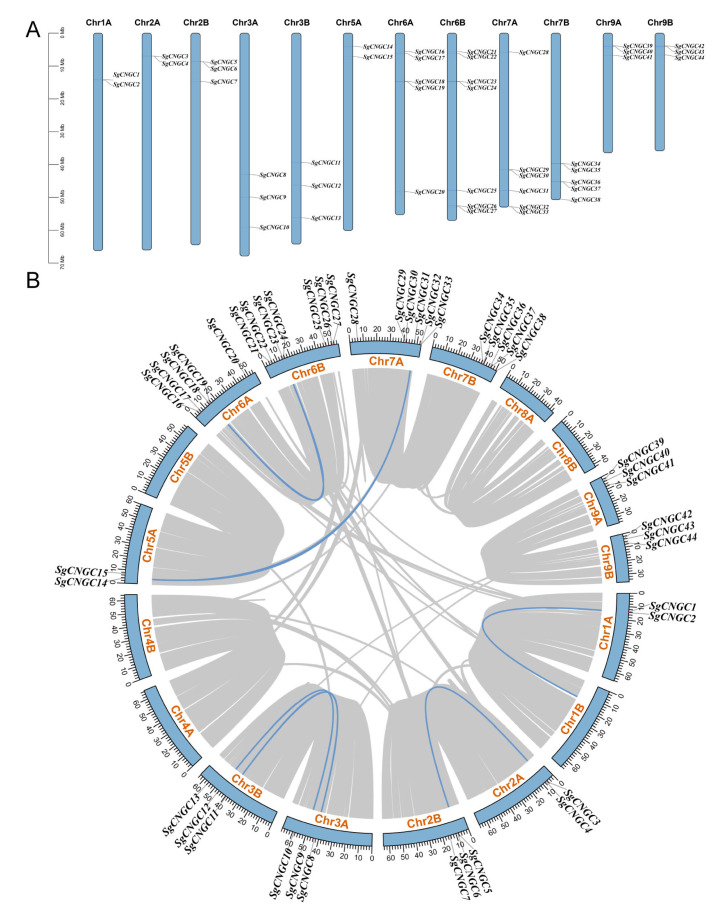
**Chromosomal localization and intraspecific collinearity of the CNGC gene family.** (**A**) The chromosome locations of chromosomes of the *SgCNGC* genes in *Suaeda glauca*. (**B**) The collinearity of *SgCNGC* genes within in *Suaeda glauca* genome, with the gray line indicating the collinear gene pairs in the genome and the blue line highlighting the collinear gene pairs within the *SgCNGC* family.

The 44 *SgCNGC* genes were unevenly distributed across 12 chromosomes (Figure 3). Chromosomes 2B, 3A, 3B, 9A, and 9B each harbored three *SgCNGC* genes, whereas the number of genes on other chromosomes ranged from 2 to 7. Chromosome 6B contained the most genes, forming a distinct gene cluster. Based on conserved regions sequence comparisons, highly similar gene sequences and tandem duplications were observed on chromosomes 2A, 2B, 6A, 7A, and 7B. Although complete gene duplication was not observed on other chromosomes, the sequences were still highly similar. Intra-species collinearity analysis of the *S. glauca CNGC* gene family revealed large-scale gene duplication events, identifying six collinear gene pairs. Among them, *SgCNGC23 & SgCNGC18*, *SgCNGC31 & SgCNGC14*, *SgCNGC4 & SgCNGC6*, *SgCNGC8 & SgCNGC11*, and *SgCNGC9 & SgCNGC12* belonged to the same subgroup, confirming closer evolutionary relationships among members of the same subgroup.

**Figure 4:**
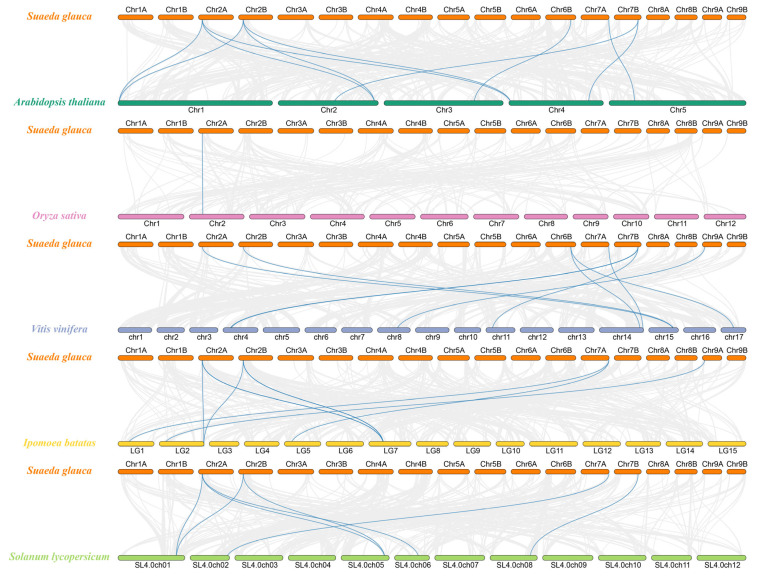
**Interspecific collinearity analysis of CNGC gene families between *Suaeda glauca* and *Arabidopsis thaliana*, *Oryza sativa*, *Vitis vinifera*, *Ipomoea batatas*, and *Solanum lycopersicum*.** Gray lines represent collinear gene pairs between the *Suaeda glauca* genome and the genomes of other plant species, while blue lines highlight collinear gene pairs of *CNGC* genes across species.

We compared *S. glauca* with both dicots and a monocot species to understand the conservation and divergence of CNGC genes during evolution. This multispecies comparison helps identify which lineages share close syntenic relationships with *S. glauca*. Collinearity analysis between *S. glauca* and five other species (*A. thaliana*, *O. sativa*, *V. vinifera*, *I. batatas*, and *S. lycopersicum*) yielded 10, 1, 8, 7, and 7 collinear gene pairs, respectively, with the highest number found with *A. thaliana* and the lowest in O. sativa (Figure 4). All dicot species formed collinear gene pairs with genes located on *S. glauca* chromosomes 2A, 2B, 7A, and 7B. These results indicate that *A. thaliana* is the most closely related model species to *S. glauca* among those analyzed.

### 3.5. Gene Structure Analysis of the S. glauca CNGC Gene Family

**Figure 5:**
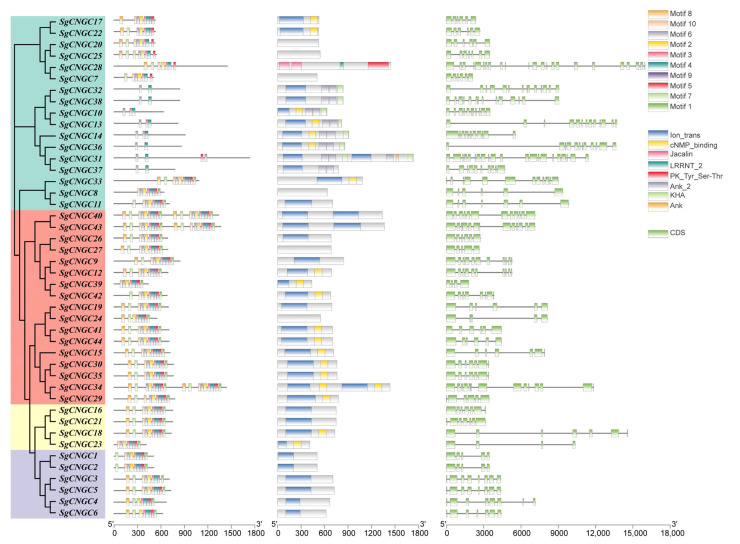
**Motif composition, conserved domains, and gene structure of the *SgCNGC* gene family.** From left to right: the phylogenetic tree, motif distribution, conserved domain architecture, and gene structure of CNGC family members. In the gene structure diagram, green rectangles represent coding sequences (CDS), and lines represent introns.

A total of 10 conserved protein motifs (Motif1–Motif10) were predicted among the 44 *SgCNGC* genes (Figure 5). Motif4 and Motif6 appeared most frequently. All *SgCNGC* genes contained Motif4, and except for *SgCNGC4*, *SgCNGC6*, *SgCNGC24*, and *SgCNGC28*, all gene possessed Motif6, indicating high conservation. Genes in subgroup Class IV contained fewer conserved motifs, suggesting that they may be more prone to mutation. Gene structure prediction identified eight conserved domains, with ion transport domains and CNBD domains being the most abundant. Additionally, CDS regions were detected in each gene. The number and length of exons and introns varied among subgroups, with members of the same subgroup showing similar exon–intron structures.

### 3.6. Cis-Acting Element Analysis Indicated That the SgCNGC Genes Might Be Involved in Drought and Salt Stress Response

**Figure 6:**
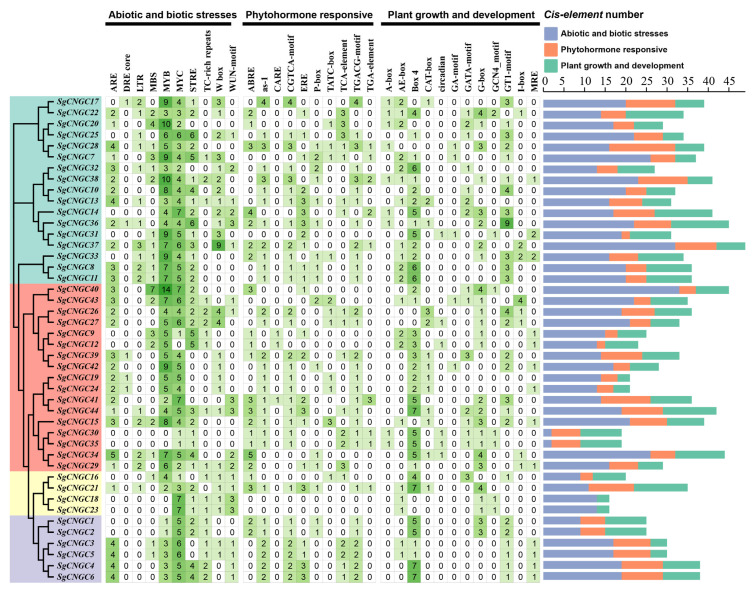
**Cis-acting elements in the promoter regions of the *Suaeda glauca* CNGC gene family.** The color intensity indicates the number of each *cis*-acting element. Elements are categorized into three functional classes, and the number per gene is displayed.

Analysis of the upstream promoter region of the SgCNGC gene (Figure 6). revealed three major categories of cis-acting elements, related to abiotic stress (e.g., drought, salt, cold, heat) and biotic stress (e.g., pathogen infection) responses, phytohormone responses, and plant growth and development, corresponding to 10, 10, and 12 elements, respectively. These included elements responsive to low temperature, auxin, abscisic acid, gibberellin, light, etc. Members of the same subgroup had similar cis-acting elements. Among elements related to abiotic and biotic stress, MYC and MYB were the most common, followed by STRE and ARE. Feng et al. [26] previously identified a significant role of the *CNGC* gene family in regulating methyl jasmonate (MeJA), salt stress, and drought stress in tomato. Furthermore, MYB and MYC, in cooperation with other transcription factors, can participate in plant salt and drought stress responses [27], implying that *SgCNGC* genes may also be involved in drought and salt stress responses, enhancing salt and drought tolerance.

### 3.7. Subcellular Localization Analysis of SgCNGC13

**Figure 7:**
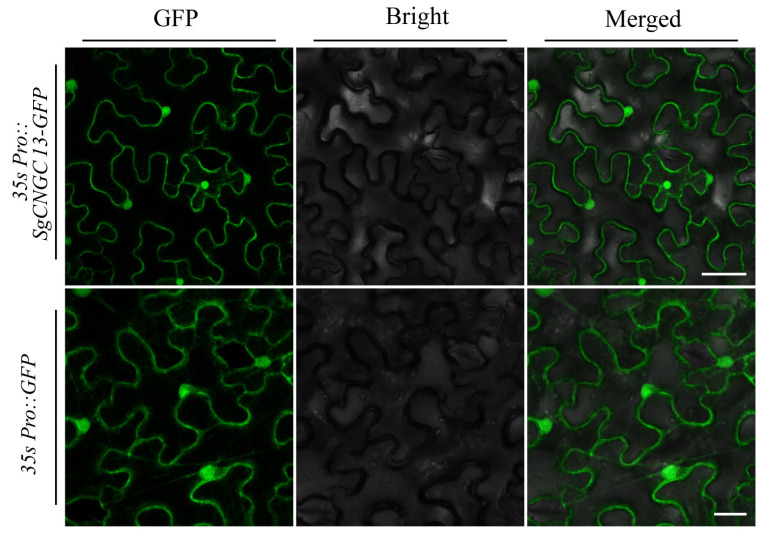
**Subcellular localization of *SgCNGC13*.** Fluorescence signals from the transient expression of the *SgCNGC13*-GFP fusion protein in *N. benthamiana* leaf epidermal cells, were compared with those from an empty vector control. Images show GFP fluorescence, brightfield, and their merged overlay (Scale bar = 20 μm).

Previous studies [14], have shown that plant *CNGC* genes are often localized to the cell membrane but also distributed in organelles such as the nucleus, cytoplasm, and chloroplasts. To further investigate the subcellular localization of *SgCNGC* genes, transient expression in *N. benthamiana* was performed to determine the localization of the *SgCNGC13*–green fluorescent protein (GFP) fusion construct, using an empty vector as a control. In the experimental group, green fluorescence signal was clearly localized to the cell periphery, highly consistent with the morphology of the cell membrane, and significant fluorescence signal was also observed in the nucleus. Overlay images of fluorescence and bright-field microscopy showed complete overlap of fluorescence signals at the cell membrane and nuclear positions with cellular structures (Figure 7).

### 3.8. Effect of SgCNGC13 Overexpression on Root Length in Transgenic A. thaliana

**Figure 8:**
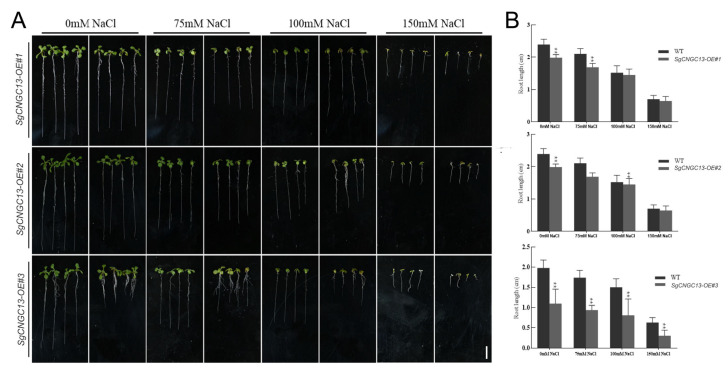
**Heterologous overexpression of *SgCNGC13* in *Arabidopsis thaliana*.** (**A**) Phenotypes of wild-type and *SgCNGC13*-overexpressing *A. thaliana* seedlings grown on 1/2 MS medium supplemented with 0, 75, 100, and 150 mM NaCl. (**B**) Statistical analysis of root lengths of wild-type and *SgCNGC13*-overexpressing *A. thaliana* under the same conditions. An ** within the same row indicates a significant difference between groups (*p* ** < 0.05) (bar = 0.5 cm).

To investigate the salt tolerance mechanisms of the *S. glauca CNGC* gene family, this study compared the growth of *SgCNGC13*-overexpressing transgenic *A. thaliana* with wild-type *A. thaliana* under different salt concentrations. Increasing salt concentrations progressively inhibited shoot and root elongation. When overexpression lines were compared with wild-type plants, no significant difference in shoot growth rate was observed. Under normal, non-stress conditions (0 mM NaCl), the average root lengths of three overexpression lines were significantly shorter than those of the wild type. These results indicates that overexpression of *SgCNGC13* affected normal plant growth and development, increasing root sensitivity. However, as salt concentration increased, the difference in root length between the two groups gradually decreased, suggesting that *SgCNGC13* overexpression may negatively regulate the plant’s salt stress response (Figure 8).

## 4. Discussion

Globally, over 1.1 billion hectares of land are affected by salinization, with crop yield reductions reaching up to 30% in certain regions [28]. Approaches such as breeding salt-tolerant crops, developing saline agriculture, and exploring the economic value of halophytes are important strategic measures for achieving sustainable development and ensuring national food and ecological security. *S. glauca* possesses unique salt-tolerant properties and serves as a “pioneer” plant for ameliorating saline–alkali soil, playing a key role in the remediation of coastal and inland saline–alkali lands in China. Therefore, research on the response of *S. glauca* genes to abiotic stresses is crucial.

This study identified 44 *SgCNGC* gene family members in *S. glauca* using bioinformatics methods. Chromosomal localization revealed their presence on only 12 out of 18 chromosomes, with an uneven distribution. The gene number is quite different from that of *A. thaliana* (20 *AtCNGC* genes) but closest to that of apple (44 *MdCNGC* genes) [29] and *Brassica napus* (49 *BnCNGC* genes), suggesting diverse roles in stress response and similarity to apple and *B. napus*. Subcellular localization prediction placed SgCNGC proteins on the cell membrane, indicating that they function as ion channel proteins, similar to the localization pattern of *A. thaliana* CNGCs [30]. Concurrently, phylogenetic analysis showed similar classification and distribution of *CNGC* gene family members between *S. glauca* and *A. thaliana*, suggesting a close evolutionary relationship and homology between the two species. Further chromosomal and collinearity analysis detected tightly clustered gene distributions at chromosome ends, representing tandem duplications. This provides a driving force for gene family expansion, helping plants adaptively evolve (e.g., resisting pathogen infection, improving stress tolerance) [31], and is one of the major means of gene amplification (tandem duplication, segmental duplication, or whole-genome duplication). However, gene duplication can also result in the loss of less important duplicate genes following polyploidization [32]. Collinearity analysis results indicated close genetic relationships between *S. glauca* and *A. thaliana*, *V. vinifera*, *I. batatas*, and *S. lycopersicum*, consistent the phylogenetic tree analysis. Fewer collinear pairs with *O. sativa* suggest that *S. glauca* shares homology with these species, but due to the dicot–monocot division, its relationship with *O. sativa* is more distant than with other dicots [33]. These findings indicate gene differentiation during plant evolution, leading to variations in gene number and structural characteristics among species. Species with similar gene numbers and collinearity patterns may also share functional similarities.

Analysis of conserved domains and sequence alignment in the *S. glauca CNGC* gene family revealed that subgroups I, II, and III were more conserved than group IV. This finding is consistent with the conclusion of Maser et al. [34], which reported that the CNGC genes in subgroup IV exhibit a more distant evolutionary relationship. Insertions and deletions of non-conserved residues were observed in subgroup IV, especially in SgCNGC13. These structural differences may directly affect the ability of proteins to bind to cyclic nucleotides, interact with calmodulins, or regulate ion fluxes, potentially leading to alterations in the stress signaling pathway. Furthermore, the high conservation of Motif4 and Motif6, along with the universal presence of ion transport and CNBD domains, confirms their essential role in channel function. The coding region structure changes with gene differentiation. Genes within the same subgroup share certain similarities in exon–intron structure (number and length of introns and exons), which influences gene expression and protein function, further validating functional similarity within subgroups [35]. Analysis of cis-acting elements provides substantial evidence supporting the potential regulatory roles of the genes. *Cis*-acting elements in the *S. glauca CNGC* gene family participate in regulating functional domains related to plant responses to abiotic and biotic stresses, phytohormone responses, and growth and development. The widespread presence of MYB and MYC binding sites directly links this gene family to the regulatory transcription that controls salt and drought stress responses.

Among the identified *S. glauca CNGC* genes, *SgCNGC13* exhibited lower conservation, possibly indicating mutation and loss of normal function. This study conducted subcellular localization of *SgCNGC13* and performed overexpression analysis in *A. thaliana*, aiming to further explore its gene function and provide insights for modifying defective genes. *SgCNGC13* was predicted to localize to the cell membrane but was found on both the cell membrane and nucleus, suggesting it primarily functions as a membrane ion channel protein while also potentially participating in nucleus-specific signaling pathways within the nucleus. Overexpression experiments suggested that *SgCNGC13* may negatively regulate salt stress responses, providing a basis for further functional analysis.

In summary, this study provides a preliminary identification and analysis of the *S. glauca CNGC* gene family, laying a theoretical foundation for future functional investigations. It also provides valuable date for subsequent analysis of salt and drought tolerance mechanisms in *S. glauca* and for the screening of stress-resistant plants. However, the specific regulatory mechanisms underlying optimized salt and drought tolerance genes in *S. glauca* require further exploration. Future research should focus on elucidating these mechanisms to provide genetic resources and technical support for promoting saline-alkali land remediation and sustainable utilization.

## 5. Conclusions

Based on the whole-genome data of *S. glauca*, this study systematically identified 44 members of the *SgCNGC* gene family. Through multi-dimensional bioinformatics analysis, the structural characteristics, evolutionary relationships, and potential functional differentiation mechanisms of these members were characterized. The results indicate a close evolutionary relationship between *S. glauca* and *A. thaliana*. Furthermore, subgroups III and IV exhibited lower conservation and more distant relationships compared to other *CNGC* gene members, suggesting potential functional differences. These genes are likely involved in salt and drought stress responses. Overexpression of *SgCNGC13* in *A. thaliana* resulted in enhanced sensitivity to salt stress, suggesting that *SgCNGC13* gene may play a negative regulatory role in salt stress responses. This study provides a valuable resource for further exploration of *S. glauca CNGC* gene functions and stress resistance breeding. However, this study has certain limitations that cannot be ignored. At present, the identification and characterization of the *SgCNGC* gene largely rely on bioinformatics prediction, and the specific regulatory mechanism of the *SgCNGC* gene underlying their functions require further experimental validation.

## Data Availability

All data and material are provided in the manuscript and Supplementary Materials.

## Supplementary Materials

The following supporting information can be downloaded at: https://www.mdpi.com/article/10.3390/biology15060467/s1, Table S1: Physicochemical properties of SgCNGC protein.
