# Supplementary material for: Genome-Wide Identification and Functional Analysis of the CNGC Gene Family in Suaeda glauca
Source: Biology (Basel). 2026 Mar 13;15(6):467. doi: 10.3390/biology15060467 (PMC13024286; doi:10.3390/biology15060467)
Supplement: Supplementary file 1 [file biology-15-00467-s001.zip › 2.Figures and legends.pdf]

Figures and legends

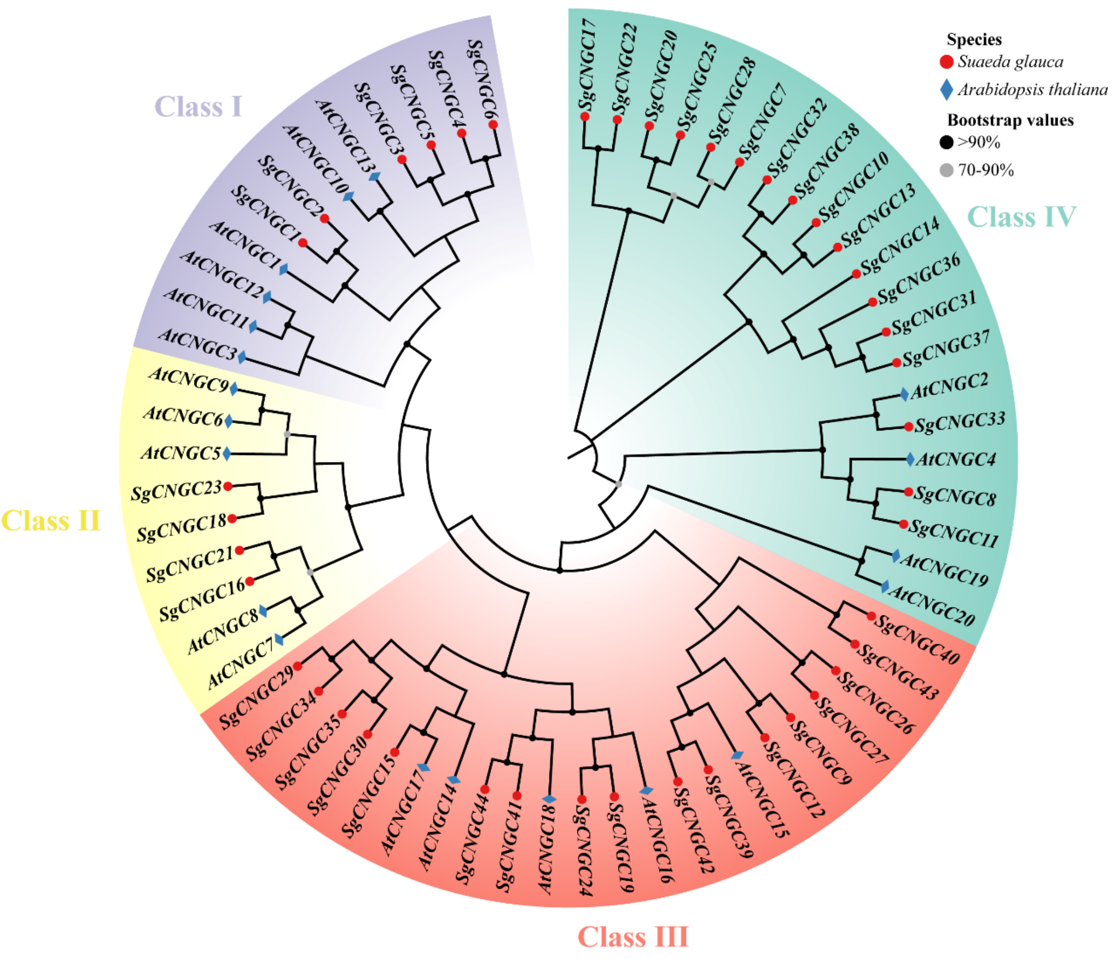

Figure 1. Phylogenetic tree of the CNGC gene family of *Suaeda glauca* and *Arabidopsis thaliana*. Purple, yellow, red and green represent Class I, II, III and IV, respectively. Red dots represent the *SgCNGC* gene family, blue squares represent the *AtCNGC* gene family. Black dots on branches indicate bootstrap values >90%, and grey indicates 70–90%.

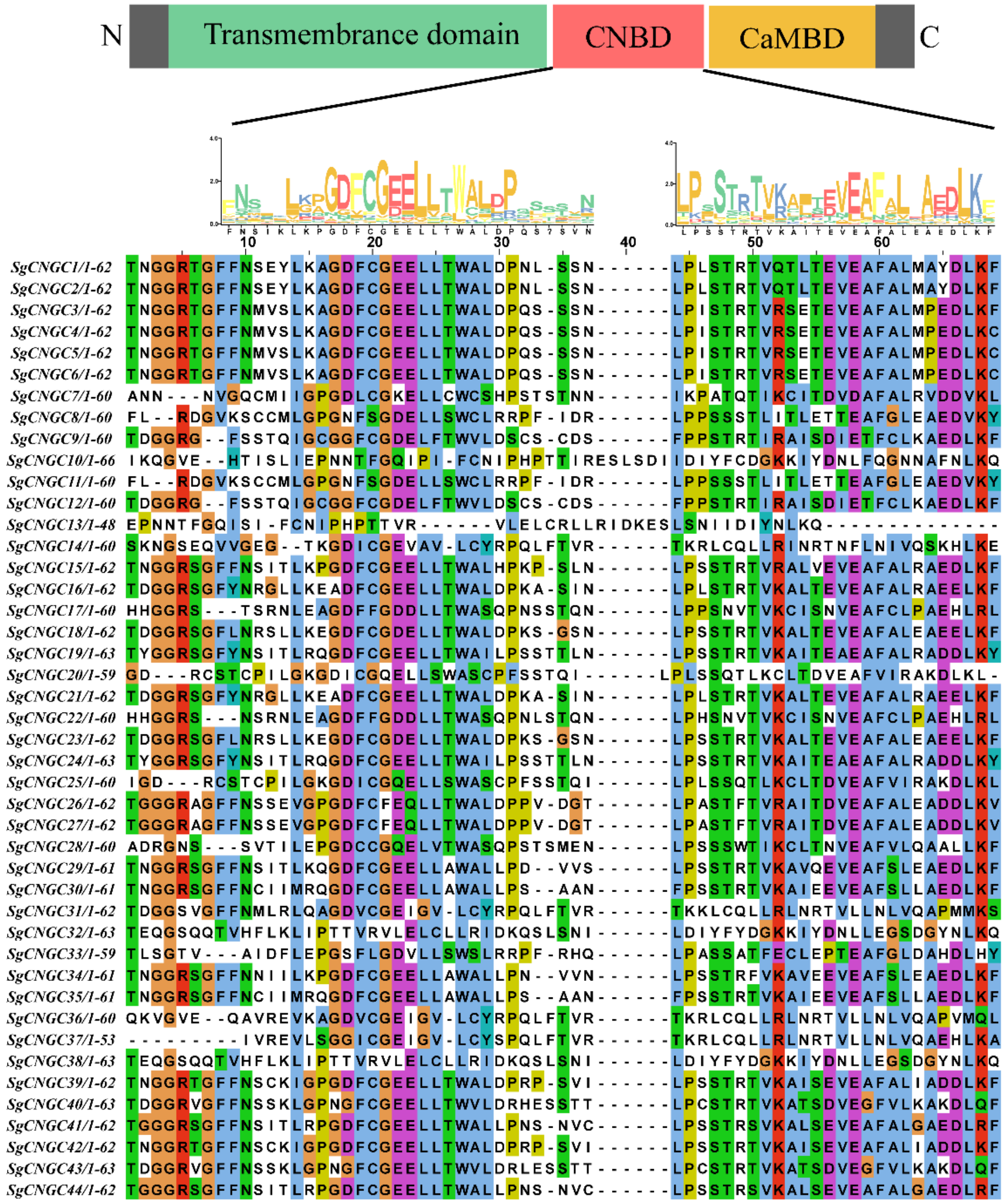

Figure 2. Conserved domains and sequence alignment of the *Suaeda glauca* CNGC gene family. Different colors represent different amino acid residues. The sequence logo at the top illustrates the conservation of residues within specific motifs in the CNBD domain, while the height of each letter is proportional to the frequency of the corresponding residue at that position.

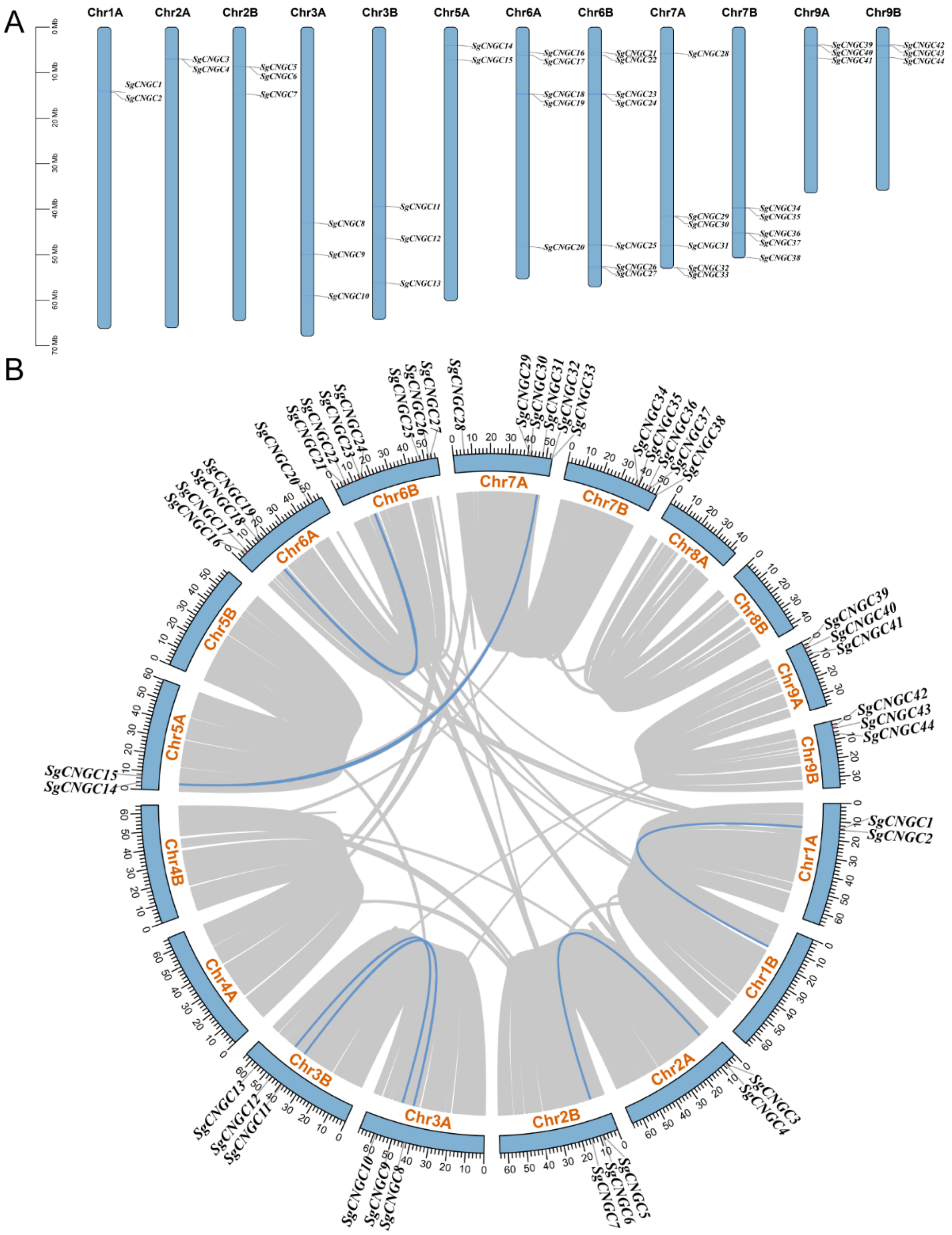

Figure 3. Chromosomal localization and intraspecific collinearity of the CNGC gene family. (A) shows the chromosome locations of chromosomes of the *SgCNGC* genes in *Suaeda glauca*. (B) shows the collinearity of *SgCNGC* genes within in *Suaeda glauca* genome, with the gray line indicating the collinear gene pairs in the genome and the blue line highlighting the collinear gene pairs within the *SgCNGC* family.

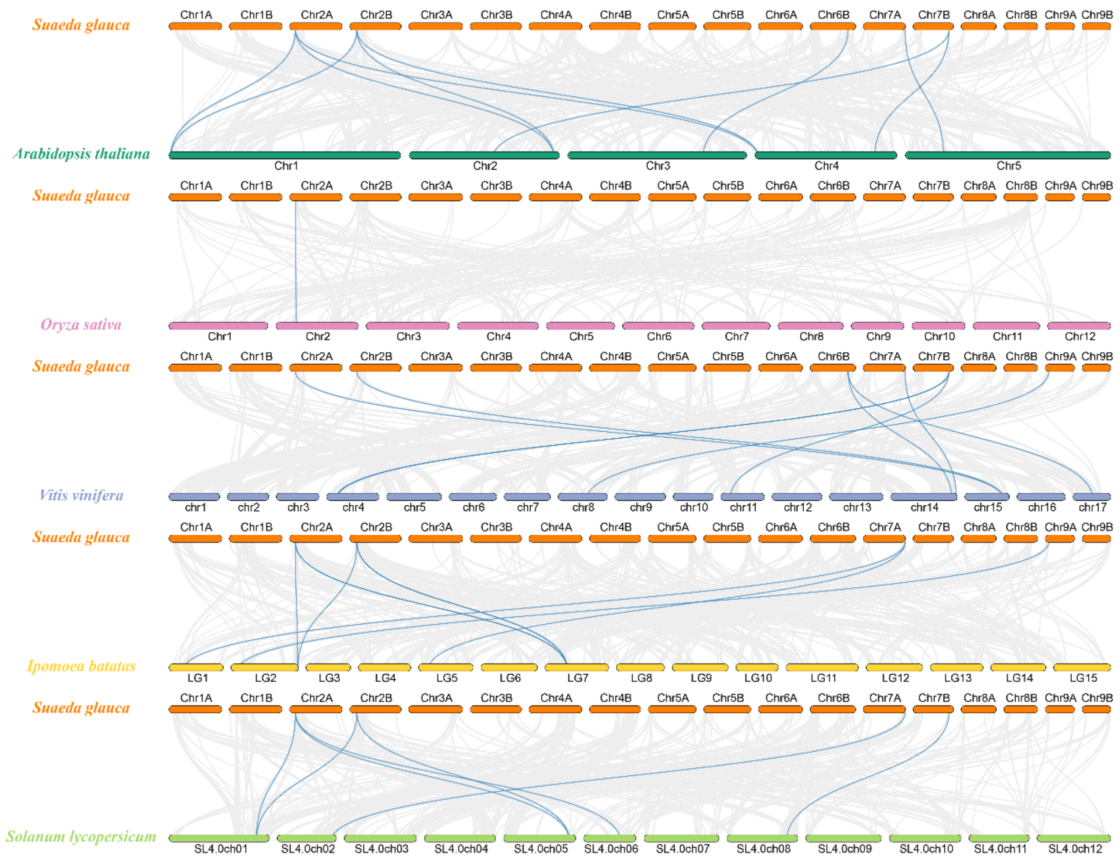

Figure 4. Interspecific collinearity analysis of CNGC gene families between *Suaeda glauca* and *Arabidopsis thaliana*, *Oryza sativa*, *Vitis vinifera*, *Ipomoea batatas*, and *Solanum lycopersicum*. Gray lines represent collinear gene pairs between the *Suaeda glauca* genome and the genomes of other plant species, while blue lines highlight collinear gene pairs of *CNGC* genes across species.

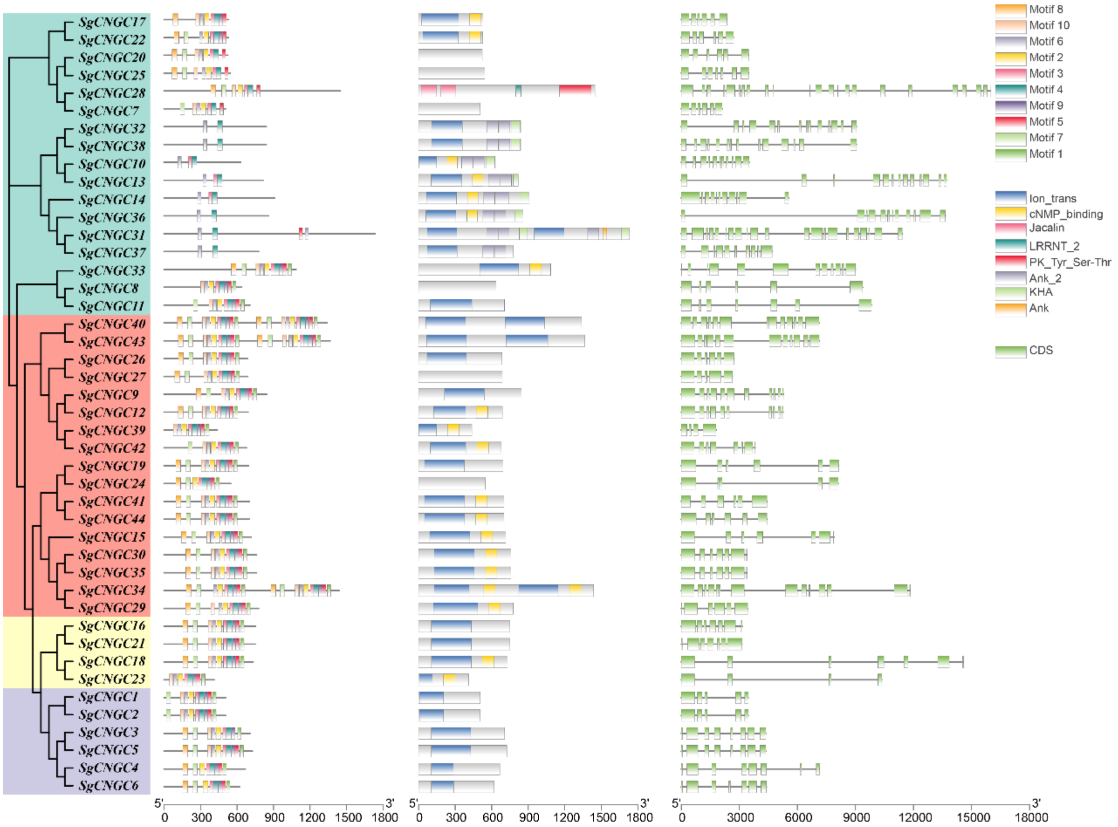

Figure 5. Motif composition, conserved domains, and gene structure of the *SgCNGC* gene family. From left to right: the phylogenetic tree, motif distribution, conserved domain architecture, and gene structure of CNGC family members. In the gene structure diagram, green rectangles represent coding sequences (CDS), and lines represent introns.

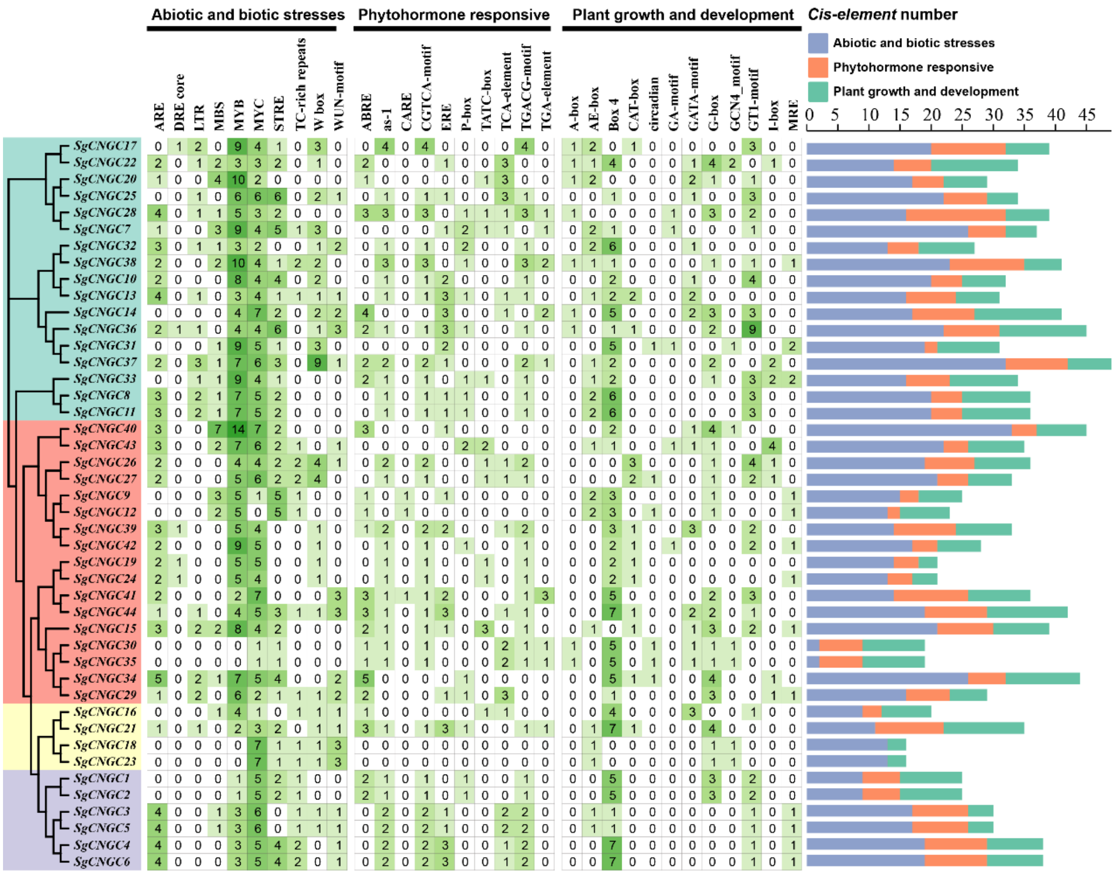

Figure 6. Cis-acting elements in the promoter regions of the *Suaeda glauca* CNGC gene family. The color intensity indicates the count of each *cis*-acting element. Elements are categorized into three functional classes, and the count per gene is displayed.

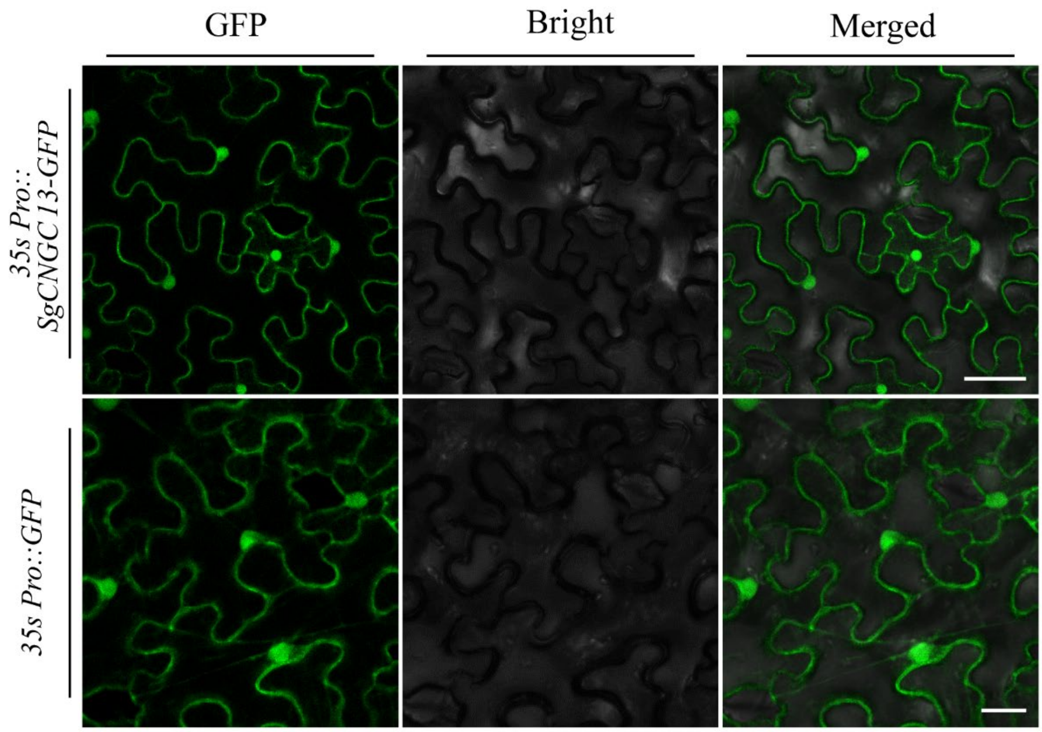

Figure 7. Subcellular localization of *SgCNGC13*. Fluorescence signals from the transient expression of the *SgCNGC13*-GFP fusion protein in *N. benthamiana* leaf epidermal cells, compared with an empty vector control. Images show GFP fluorescence, brightfield, and their merged overlay (Bar = 20 μm).

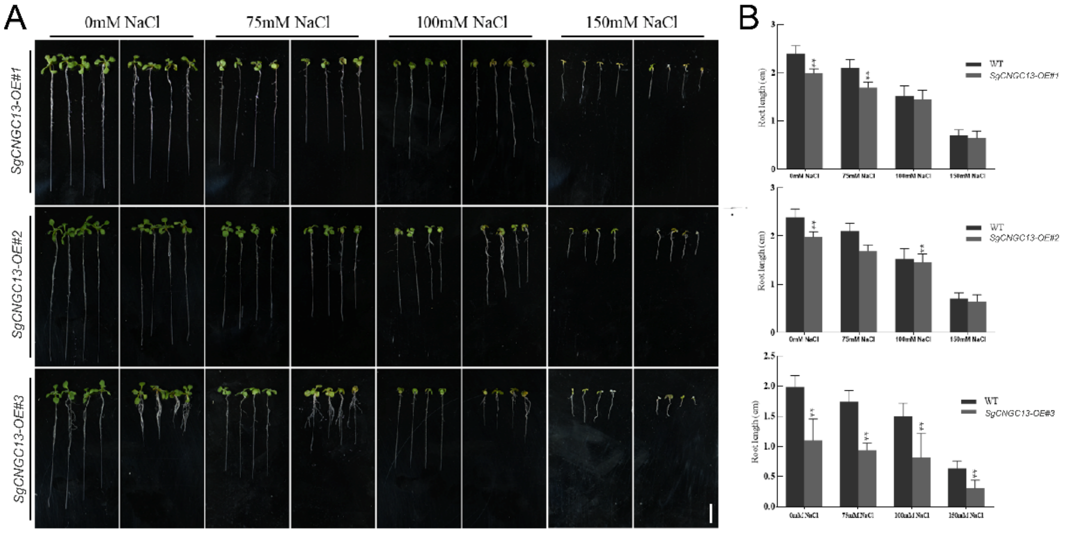

Figure 8. Heterogenous over-expression of *SgCNGC13* in *Arabidopsis thaliana*. (A) Phenotypes of wild-type and *SgCNGC13*-overexpressing *A. thaliana* seedlings grown on 1/2 MS medium supplemented with 0, 75, 100, and 150 mM NaCl. (B) Statistical analysis of root lengths of wild-type and *SgCNGC13*-overexpressing *A. thaliana* under the same conditions. * Within the same row indicates a significant difference between groups ($P^* < 0.05$). (Bar = 0.5 cm).
