# Supplementary material for: Genome-Wide Identification and Functional Analysis of the CNGC Gene Family in Suaeda glauca
Source: Biology (Basel). 2026 Mar 13;15(6):467. doi: 10.3390/biology15060467 (PMC13024286; doi:10.3390/biology15060467)
Supplement: Supplementary file 1 [file biology-15-00467-s001.zip › 3.Supplemental Tables.pdf]

Table S1 Physicochemical properties of SgCNGC protein.

Gene ID	Gene name	Amino acid/aa	Molecular weight/kDa	pI	Aliphatic index	Subcellular localization
Sg.1G0000941a	*SgCNGC1*	505	58.8	8.76	83.09	Plasma Membrane
Sg.1G0000941b	*SgCNGC2*	505	58.8	8.76	83.09	Plasma Membrane
Sg.2G0000713a	*SgCNGC3*	706	81.07	9.05	84.67	Plasma Membrane
Sg.2G0000715a	*SgCNGC4*	667	77.15	9.8	80.72	Plasma Membrane
Sg.2G0000713b	*SgCNGC5*	725	83.39	9.24	84.21	Plasma Membrane
Sg.2G0000715b	*SgCNGC6*	619	70.87	9.56	86.8	Plasma Membrane
Sg.2G0000510a	*SgCNGC7*	505	56.84	8.83	96.75	Plasma Membrane
Sg.3G0001157a	*SgCNGC8*	637	73.65	8.56	86.94	Plasma Membrane
Sg.3G0001757a	*SgCNGC9*	844	97.53	6.03	88	Plasma Membrane
Sg.3G0002709a	*SgCNGC10*	630	71.67	6.48	98.13	Plasma Membrane
Sg.3G0001157b	*SgCNGC11*	705	81.77	8.9	90.99	Plasma Membrane
Sg.3G0001757b	*SgCNGC12*	689	79.6	5.76	88.56	Plasma Membrane
Sg.3G0002709b	*SgCNGC13*	817	93.88	7.86	95.08	Plasma Membrane
Sg.5G0000410a	*SgCNGC14*	909	102.95	6.5	93.89	Plasma Membrane
Sg.5G0000678a	*SgCNGC15*	715	82.05	9.24	93.97	Plasma Membrane
Sg.6G0000565a	*SgCNGC16*	749	85.85	9.09	89.97	Plasma Membrane
Sg.6G0000630a	*SgCNGC17*	529	60.61	8.68	106.48	Plasma Membrane
Sg.6G0001221a	*SgCNGC18*	731	83.76	8.99	91.27	Plasma Membrane
Sg.6G0001223a	*SgCNGC19*	694	81.22	8.95	86.71	Plasma Membrane
Sg.6G0002727a	*SgCNGC20*	523	60.05	6.28	106.06	Plasma Membrane

Sg.6G0000565b	*SgCNGC21*	749	85.85	9.09	89.97	Plasma Membrane
Sg.6G0000630b	*SgCNGC22*	526	59.47	8.82	107.66	Plasma Membrane
Sg.6G0001221b	*SgCNGC23*	411	47.6	8.79	81.92	Plasma Membrane
Sg.6G0001223b	*SgCNGC24*	547	64.09	9.47	88.28	Plasma Membrane
Sg.6G0002727b	*SgCNGC25*	544	62.21	6.25	103.58	Plasma Membrane
Sg.6G0002802b	*SgCNGC26*	684	79.19	6.58	95.66	Plasma Membrane
Sg.6G0002769b	*SgCNGC27*	687	79.29	6.99	94.38	Plasma Membrane
Sg.7G0000680a	*SgCNGC28*	1448	162.43	6.8	86.73	Plasma Membrane
Sg.7G0001939a	*SgCNGC29*	776	89.88	9.05	85.94	Plasma Membrane
Sg.7G0001961a	*SgCNGC30*	759	87.7	9.56	84.8	Plasma Membrane
Sg.7G0002267a	*SgCNGC31*	1734	194.87	6.48	98.1	Plasma Membrane
Sg.7G0002660a	*SgCNGC32*	839	96.68	6.54	94.35	Plasma Membrane
Sg.7G0002663a	*SgCNGC33*	1084	123.28	9.53	93.83	Plasma Membrane
Sg.7G0001939b	*SgCNGC34*	1437	166.15	9.15	85.84	Plasma Membrane
Sg.7G0001961b	*SgCNGC35*	759	87.7	9.56	84.8	Plasma Membrane
Sg.7G0002262b	*SgCNGC36*	859	96.6	6.23	100.42	Plasma Membrane
Sg.7G0002267b	*SgCNGC37*	777	87.65	6.71	97.62	Plasma Membrane
Sg.7G0002660b	*SgCNGC38*	839	96.68	6.54	94.35	Plasma Membrane
Sg.9G0000377a	*SgCNGC39*	437	50.67	8.37	84.83	Plasma Membrane
Sg.9G0000393a	*SgCNGC40*	1338	154.68	8.68	92.89	Plasma Membrane
Sg.9G0000532a	*SgCNGC41*	701	81.14	8.97	86.66	Plasma Membrane
Sg.9G0000377b	*SgCNGC42*	678	77.56	8.63	91.62	Plasma Membrane

Sg.9G0000393b	*SgCNGC43*	1364	157.53	8.75	92.69	Plasma Membrane
Sg.9G0000532b	*SgCNGC44*	701	81.14	8.97	86.66	Plasma Membrane
